# Essential newborn care practices for healthy newborns at a district hospital in Pemba, Tanzania: a cross-sectional observational study utilizing video recordings

**DOI:** 10.1080/16549716.2022.2067398

**Published:** 2022-06-08

**Authors:** Christina Nadia Stensgaard, Christine Manich Bech, Charlotte Holm-Hansen, Tine Bruhn Skytte, Said Mohammed Ali, Ulfat Amour Mohd, Jesper Kjærgaard, Gorm Greisen, Anja Poulsen, Stine Lund

**Affiliations:** a Global Health Unit, Department of Paediatrics and Adolescent Medicine, The Juliane Marie Centre, Copenhagen University Hospital Rigshospitalet, Copenhagen, Denmark; b Public Health Laboratory Ivo de Carneri, Pemba, Tanzania; c Department of Neonatology, The Juliane Marie Centre, Copenhagen University Hospital Rigshospitalet, Copenhagen, Denmark

**Keywords:** ENC interventions, ENC compliance, WHO recommendations, low resource setting, video observations, Hawthorne effect

## Abstract

**Background:**

High-quality essential newborn care (ENC) can improve newborn health and reduce preventable newborn mortality. The World Health Organization recommends specific ENC interventions. Video recordings have potential as a tool for assessment of clinical care also in low and middle-income countries.

**Objective:**

To use video observations of healthy newborns to describe ENC practices in a low-income setting and compare actual clinical practice with WHO recommendations.

**Method:**

This is a cross-sectional observational study. Video records of neonatal interventions to 324 healthy newborns were assessed. They were obtained at baseline of a pre-post intervention study during a 10-week study period in Pemba, Tanzania. Data also included postnatal structured questionnaires. Eight ENC interventions and quality indicators were defined as per the WHO recommendations. Descriptive statistics were used to summarize ENC practices and maternal and neonatal characteristics.

**Results:**

None of the newborns received all eight recommended ENC interventions. The median duration of separation from the mother was 25 minutes and 15 seconds (ranging from 22 seconds to 3 hours and 36 minutes), 51% of the newborns received proper thermal care during the separation. Twenty-one percent had sufficient umbilical cord care, 8% were stimulated for breathing, 69% were observed at least once by healthcare staff and 9% did undergo suctioning. None of the newborns received antibiotic ointments or vitamin K.

**Conclusion:**

Video recording of healthy newborns was feasible. The study identified omission of key ENC practices including proper thermal care, skin-to-skin contact and establishment of breastfeeding within the first hour of life, vitamin K administration as well as application of unnecessary practices such as excessive suctioning of breathing newborns.

## Background

The rate of neonatal mortality decreased by 51% between 1990 and 2017 [1]. Despite this, annually, 2.9 million newborns die during the first 28 days of their lives [2]. The reduction in annual neonatal mortality, of approximately 2% since publication of the Millennium Development Goals in 1990, lagged behind the decrease in the rate of under-five mortality during the same period [3]. Prioritizing the health of newborns is on the global agenda, and the United Nations’ sustainable development goal (SDG) 3 is to secure ‘reduced neonatal mortality to at least as low as 12 per 1,000 live births’ by 2030 [4]. It is estimated that 71% of neonatal deaths could be avoided by 2025, together with high healthcare coverage [3]. Two-thirds of countries at risk of failing to meet the SDG target for neonatal mortality are situated in sub-Saharan Africa, which makes it the region with the greatest challenge in this regard [1].

The World Health Organization (WHO) recommends essential newborn care (ENC) interventions to secure an early diagnosis and prevent illness in newborns [5]. A key recommendation is that healthy newborns should be placed skin to skin with their mother during the first hour of birth or wrapped and sufficiently covered if separated. Breastfeeding should be established as soon as possible after birth and within the first hour. Newborns should only undergo suction when it is clearly indicated and should be under surveillance and evaluated for danger signs [5]. The most effective impacts of ENC practices in aversion of newborn morbidity and mortality relate to thermal care, skin-to-skin contact and breastfeeding [6–10].

Existing research has primarily focused on newborns with low birthweight or illness [3]. There is little insight into ENC practices for healthy newborns in the literature, and evidence is based on direct observations and data extracted from medical files [11–15]. Video recordings were first used to analyse neonatal resuscitation scenarios in 1999 [16]; since then, only a few studies have used this approach to assess delivery room practices in low- and middle-income countries [17,18]. To the best of our knowledge, this is the first study that use video observations to describe and assess quality of care in ENC practices of healthy newborns in a low-income setting

## Methodology

### Study design

This cross-sectional observational study drew on data obtained during the baseline period of a pre-post intervention ‘Newborn Emergency Outcome study’ (clinicaltrials.gov:NCT04093778, Zanzibar Health Research Institute: NO.ZAHREC/2 August 2019/30) in Pemba, Tanzania with the aim of reducing neonatal mortality. Motion-triggered video cameras mounted on top of resuscitation tables in the delivery room of each district hospital were used to collect data presented in this paper during a 10-week period from 13 September 2019 to 22 November 2019. The Strengthening the Reporting of Observational studies in Epidemiology checklist was used to apply the methodology used in the current study.

### Setting

Pemba has a population of about 400,000 and four district hospitals [19]. This study included data collected at Chake Chake District Hospital, a secondary level hospital with a catchment population of about 100,000 [19] and with approximately 4–5,000 annual deliveries [20]. The stillbirth rate is estimated at 27.7 per 1,000 live births, and the neonatal mortality rate is approximately 16 per 1,000 live births [21]. There are no tertiary hospitals in Pemba, so in case of an emergency transfer is made to the closest tertiary hospital in Unguja which is reached by air or ferry. The main delivery room has three delivery beds and one resuscitation table. In addition, the hospital has a movable table for resuscitations in the operating theatre. The resuscitation tables are also used for the post-delivery observations of healthy newborns not undergoing resuscitation. At Chake Chake Hospital, midwives are responsible for the postnatal care of all neonates, with 3–4 midwifes at work during daytime and 2 during the night. The available equipment for ENC care consists of gloves, umbilical cord clamps, stethoscopes, oximeters, and bulb suction. Furthermore, a traditional cloth called a Kanga was brought by the women for wrapping, drying, and securing sufficient thermal care for the newborn after delivery.

### Participants

Healthy newborns delivered at Chake Chake District Hospital during the 10-week study period and their mothers were eligible for participation. Newborns undergoing neonatal resuscitation or showing danger signs were included in a different analysis, and these data will be presented elsewhere. Prior to the delivery the participants provided written consent to participate in the study. Women in the maternity and delivery wards were enrolled as soon as possible after admission; until the expulsion phase of the second stage of labour. Women in an obstetric emergency or with late presentation was not approached. After delivery consent could be obtained for the postnatal questionnaire. Research assistants asked the women for informed consent to video record the post-delivery care of their newborns and to complete a postnatal questionnaire, which was completed in cooperation with them. The research assistants were present in the maternity and delivery wards 24 hours a day to enrol women in the study, obtain their consent and complete the postnatal questionnaire. The health worker in charge of each birth, assisted by a research assistant, also completed a postnatal questionnaire as soon as possible after the delivery. Prior to the beginning of the study period the healthcare workers provided their consent to participate.

### Data sources and management

A motion-triggered camera (Oco II Smart Cam Pro®, Oco Group Inc., Irvine, California, USA) was used to record the videos; it was installed on top of the resuscitation table and recorded all instances of newborns being placed on the table. The newborn was placed on the resuscitation table immediate after the health worker cut the umbilical cord and before active management of third stage of labour. A research assistant would cover the camera if any woman, who did not provide consent, was in the delivery room since the table was shared. Only newborns, the resuscitation table with surrounding floor and the hands and feet of the healthcare provider were in the field of vision to ensure the confidentiality of staff. The parents, obstetric procedures and caregivers faces where not visible. The research assistant placed each individually assigned identification card on the resuscitation table prior to or after placement of the newborn. The videos contained time stamps and identification numbers. The identification number was applied to each woman and her newborn until discharge. The recorded videos were stored on an encrypted micro-SD card in the camera, and the data were uploaded to a secure database that could only be assessed by the international PI with an encryption code and key. All videos are deleted after the final analyses from the study. The video recordings were only for research purposes and only the international study team had access to the video recordings to ensure the individual health workers’ anonymity. The postnatal questionnaires and data on the participants’ sociodemographic, obstetric, delivery and neonatal characteristics were collected and directly entered into Lenovo® (version 7) tablets using secure data collection software (i.e. RedCap® version 5.12.1). Birthweight was cross matched with the data from the hospital registers owing to inconsistencies for a small number of observations.

### Definitions and variables

Eight WHO essential newborn care interventions were observed on the video recordings. For each ENC indicator corresponding actions in video recordings i.e. video indicators and their subcategories, were defined by a team comprising two doctors specialized in paediatrics and a paediatric resident (Table 1) [5]. Some ENC recommendations like immediate drying, removing of the wet towel, APGAR scores, administration of vaccines and weighing of the newborns were not included in the analysis because they were out of view of the camera and/or most likely happened either before the newborn was placed on the table or after it was removed.

**Table 1.** Definition of essential newborn care indicators and their corresponding actions in video recordings

WHO Essential Newborn Care indicator	Video indicators	Video indicators subcategories
1. Thermal care		
The newborn should be wrapped and covered sufficiently	Newborn wrapped and head covered	Not covered when placed on table
		Incompletely covered when placed on table
		Uncovered while on table
		Fully covered while on table
Avoid separating the newborn from its mother and keep skin-to-skin contact	Separation time from mother = total time on resuscitation table	
2. Stimulation		
Newborns should be stimulated correctly	Number of times newborn is stimulated. Stimulation defined as rubbing the newborn’s back.	
3. Routine suctioning		
Newborns without need of ventilation should not undergo oral or nasal suction	Newborns without need of ventilation who did not undergo suction	
	Total time spent on suction	Total suction time
	Oral vs nasal suction and number of suction events	Oral suction, number of events
		Nasal suction, number of events
	Correct suctioning technique defined as air pushed out of the suction bulb before placement in nose/mouth and then released	Yes
		No
		Uncertain
4. Observation		
Newborns should not be left unattended and should be check for breathing and colour.	Number of times the newborn was assessed by health worker	Visually assessed*
		Clinical examination**
		Assessed with stethoscope
5. Umbilical cord care		
The cord should be checked for bleeding, kept clean and dry. If bleeding, retied	Number of times abdomen is assessed with unwrapping and the cord retied or clamped if needed.	Cord checked
		Cord clamped
6. Vitamin K		
Newborns should be given 1 mg of vitamin K after delivery	Newborns receiving intramuscular administration of vitamin K	
7. Antibiotic eye ointment		
Newborns should receive antibiotic ointment	Newborns where application of antibiotic ointment was done	
8. Breastfeeding		
Newborns should be breastfed within the first hour after birth	Separation time from mother = total time on resuscitation table <1 hour the first hour	

*Note: Defined as healthcare worker observing the newborn, with no other interactions

**Note: Defined as uncovering of the newborn’s chest for examination of breathing and colour

### Data analysis

The video recordings were analysed according to the ENC video indicators defined in Table 1 by two independent researchers. In the event of a lack of consensus, the researchers consulted another member of the study team. The evaluation took place from the placement of the newborn on the resuscitation table and considered subsequent events until the newborn was removed from the table, as per the ENC video indicators and subcategories (Table 1). Data from the video observations were transferred from Excel® (2011 version) (Microsoft® Corporation, Redmond, Washington, USA), and the quantitative variable data were transferred from REDcap®, to SPSS® (version 27) (IBM, New York, USA) to perform descriptive statistical analysis. The continuous variables were categorised according to common medical standards and the risk factors for newborns. The median time for each quality indicator was calculated and presented as the minimum/maximum period and interquartile range (IQR). The number of observations for each qualitative indicator per newborn was grouped and specified. The indicators were furthermore compared with number of births per week. To determine any correlation between the quality indicators, a two-sample *t*-test was performed. Video indicators regarding suctioning, stimulation and administration of vitamin K and antibiotic eye ointment were afterwards compared with the postnatal questionnaires to account for findings possibly not captured on video.

## Results

**Figure 1.**
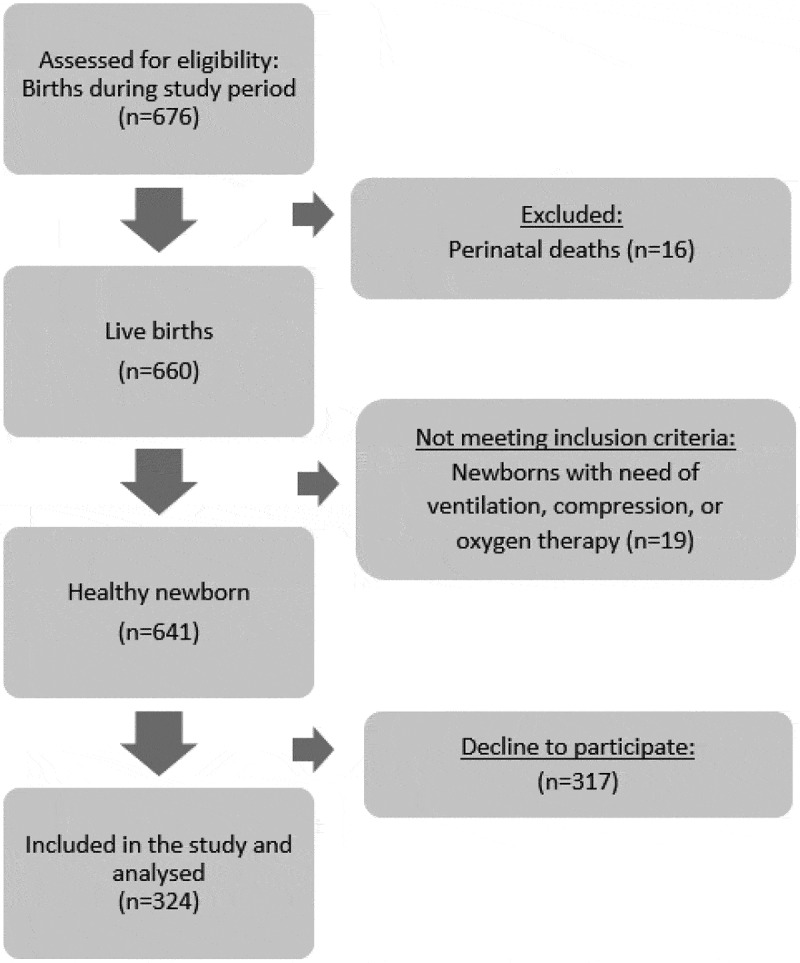
Flowchart of study population.

During the study period, 676 deliveries took place at Chake Chake District Hospital. Sixteen newborns died, 19 newborns were excluded owing to the need for neonatal resuscitation, and 317 newborns did not have video recordings. Of the 676 deliveries, 547 (81%) newborns had a postnatal questionnaire completed. Ultimately, 324 healthy newborns were eligible for inclusion in the ENC video analysis (Figure 1). Three hundred and one newborns were linked with individual identification numbers to the structured questionnaire. The questionnaire-based data for the remaining 23 newborns were not collected; however, their videos were included in the analysis. Video recording of newborns placed at the resuscitation table was practical and technical feasible and well accepted by both parents and health workers as described in a soon to be published paper from the NEO-study. The study showed that 91% of the women and 96% of health care staff was either comfortable or very comfortable with the video recordings [22].

### Socioeconomic, obstetric and delivery characteristics

The median maternal age of the participants was 26 years (IQR of 22–30 years); 18% of the pregnant women were aged ≤ 20 years. Most women were married (97%) and housewives (82%). As a minimum, three quarters of the women (77%) had finished secondary school. Median parity on admission was 3 (IQR of 2–5); 25% of the women were primigravida. Only 47% of them could provide an estimated gestational age; there were no reports of gestational age of < 37 weeks. There were no newborns with birthweight of < 1,500 g; 11% had a birthweight of < 2,500 g. The mean birthweight was 3.2 kg (standard deviation = 0.47 kg). The participants’ socioeconomic, obstetric and delivery characteristics are depicted in Table 2.

**Table 2.** Socio-demographics, obstetric, and delivery characteristics of women and their healthy newborns

	Participants n (%)*
Maternal age	
<20	53 (18%)
20–34	208 (69%)
>35	40 (13%)
Religion	
Muslim	296 (98%)
Christian	5 (2%)
Other	0 (0%)
Marital status	
Married	292 (97%)
Single	7 (2%)
Divorced	2 (1%)
Highest level of education	
No formal education	24 (8%)
Primary	45 (15%)
Secondary	213 (71%)
College	9 (3%)
University	10 (3%)
Occupation	
Housewife	248 (82%)
Farmer	5 (2%)
Government employed	20 (7%)
Self-employed	30 (10%)
Unemployed	1 (0%)
Parity on admission	
Para 0	74 (25%)
Para 1–4	169 (56%)
Para >5	58 (19%)
Antenatal care (ANC) during this pregnancy	
1–3 ANC visits	160 (53%)
>4 ANC visits	136(45%)
None	1(0%)
Missing	4(1%)
Estimated gestational age**	
<37 weeks	0 (0%)
>37 weeks	141 (47%)
Missing	160 (53%)
Mode of delivery	
Vaginal delivery	283 (94%)
Cesarean section	17 (6%)
Missing	1 (0%)
Presentation of baby	
Cephalic	295 (98%)
Breech	5 (2%)
Missing	1 (0%)
Birth weight	
<1500 g	0 (0%)
1500–2500 g	32 (11%)
2500–4000 g	251 (83%)
>4000 g	7 (2%)
Missing	11 (4%)
Sex	
Male	150 (50%)
Female	151 (50%)
Multiple gestation	
Singletons	295 (98%)
Twins	6 (2%)
*23 missing cases ** The women included provided an estimated gestational age in the postnatal questionnaire	

### Essential newborn care

The eight defined ENC indicators and their subcategories were used to analyse each video of the newborns (Table 3). Of the 324 newborns, 167 (51%) were fully covered while on the resuscitation table. One hundred and six newborns (33%) uncovered themselves from a loosely wrapped cover while on the resuscitation table and were left exposed for a median time of 10 minutes and 29 seconds (range 31 seconds to 1 hour and 42 minutes). Twenty-five newborns (8%) were observed to be stimulated on video with reports of 113 (38%) being stimulated in the postnatal questionnaire. Twenty-eight healthy breathing newborns observed on video (9%) and twenty-nine in the postnatal questionnaire without any apparent need for suctioning underwent suction with a nasal aspirator attached to a reusable suction bulb (Penguin Newborn Suction®, Laerdal Medical, Stavanger, Norway). Average total suction time was 1.5 minutes (ranging from five seconds to eight minutes). Thirteen (48%) of the 28 newborns who underwent suctioning experienced more than five events, including both oral and nasal suctioning. Eight newborns (29%) were suctioned with an incorrect suctioning technique. Specifically, the suction device was compressed after being placed inside the oral or nasal cavity, thereby blowing secretions into the newborn instead of removing them. Ten of the 25 newborns who were stimulated underwent suction as well. No correlation between the number of suction events and stimulation, associated with respiratory insufficiency, was observed (*p* = > 0.700).

**Table 3.** Essential newborn care intervention compliance by video indicator subcategories

Video indicators	Video indicators subcategories	Number of events	Number of newborns n = 324	Median time hour:min:second (min/max)	IQR hour:min:second
1. Thermal care					
Newborn wrapped in kanga and head covered	Not covered when placed on table		76 (23%)	00:02:11 (00:00:17/00:38:49)	00:08:28
	Uncovered while on table		106 (33%)	00:10:29 (00:00:31/01:42:11)	00:14:19
	Fully covered the whole time spent on table		167 (51%)		
Separation time = total time on resuscitation table	Separation time		324	00:25:15 (00:00:22/03:35:58)	00:25:10
2. Stimulation					
Newborn stimulated correctly			25 (8%)		
3. Routine suctioning					
Newborns who did not undergo suction			296 (91%)		
Suction time	Total suction time		28 (9%)	00:01:09 (00:00:05/00:08:04)	00:01:33
Place	Oral suction	1	2 (1%)		
		2–5	8 (3%)		
		>5	12 (4%)		
	Nasal suction	1	3 (1%)		
		2–5	12 (4%)		
		>5	4 (1%)		
Correct suctioning technique	Yes		11 (39%)		
	No		8 (29%)		
	Uncertain		9 (32%)		
4. Observation					
Newborn assessed	Visually assessed	0	99 (31%)		
		1	101 (31%)		
		>1	124 (38%)		
	Clinical examination	1	30 (9%)		
		>1	7 (2%)		
	Assessed with stethoscope	1	2 (1%)		
5. Umbilical cord care					
Cord checked	Cord checked	1	59 (18%)		
		>1	9 (3%)		
Retied if bleeding**	Cord clamp	1	13 (4%)		
		>1	2 (1%)		
6. Vitamin K					
Intramuscular administration of vitamin K			0 (0%)		
7. Antibiotic eye ointment					
Application of antibiotic eye ointment			1 (0%)		
8. Breastfeeding					
Separation time from mother<1 hour			293 (90%)		
Separation time from mother>1 hour			31 (10%)	01:17:44 (01:00:22/03:35:58)	00:41:34
**Note: there were no observations of bleeding from the cord, every observation was adding of an extra cord clamp and shortening of the umbilical cord.					

Ninety-nine newborns (31%) were not evaluated for colour or breathing by the healthcare staff at the resuscitation table; 101 newborns (31%) were assessed once, and 124 newborns (38%) were checked more than once. Of the 37 newborns who were clinically examined, 30 (9%) were examined once and 7 (2%) twice or more. Two newborns (1%) were assessed using a stethoscope. Sixty-eight newborns (21%) had their cord checked for bleeding, and an extra cord clamp was applied 15 times (5%). Bleeding was not observed from the cord in any cases, and extra cord clamps were used to shorten the umbilical cord. None of the newborns received the intramuscular administration of vitamin K, and one newborn was administered antibiotic eye ointment. This corresponds to the findings in the postnatal questionnaires, with no reports of vitamin K or antibiotic eye ointments administration.

The time spent on the resuscitation table (25 minutes and 15 seconds on average, range 22 seconds to 3 hours and 36 minutes) was used as a surrogate marker for the duration of separation from their mothers. Thirty-one newborns (10%) spent more than one hour on the resuscitation table; therefore, they did not meet the recommendation of breastfeeding within an hour of being born. One newborn who was delivered by Caesarean section was fed by cup while on the resuscitation table and spent 30 minutes separated from its mother. No other newborns were fed while on the resuscitation table.

**Figure 2.**
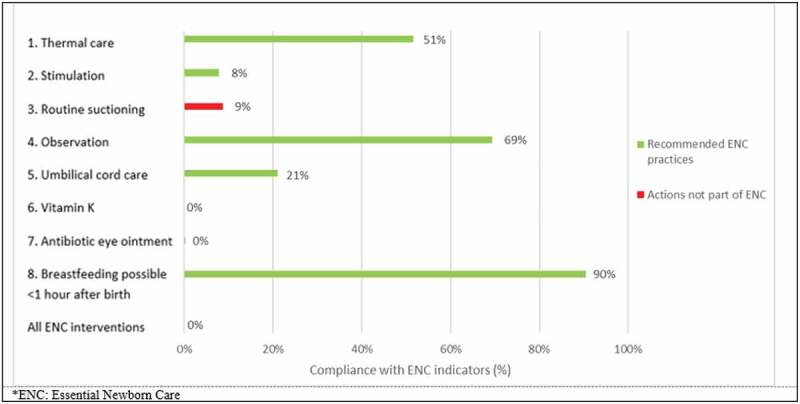
Essential newborn care compliance to WHO indicators at a Tanzanian referral hospital.

**Figure 3.**
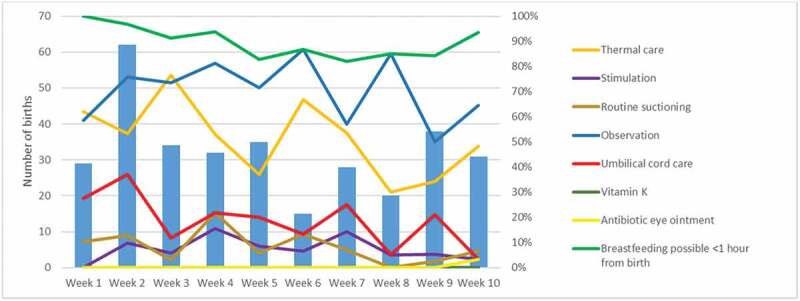
Number of births per week compared with compliance to ENC indicators at a Tanzanian referral hospital.

None of the newborns received all eight ENC interventions (Figure 2). Less than half of the newborns were stimulated, received sufficient umbilical cord care, vitamin K and antibiotic eye ointment. More than half of them received proper thermal care and were observed by the healthcare staff while separated from their mother. One out of 10 healthy breathing newborns did undergo suctioning, which are not recommended by the WHO. In addition, 90% of the newborns could have started breastfeeding within an hour. However, determining this fell beyond the scope of the current study. When analysing the results in a weekly manner the only change in practice with number of births is the observation indicator. In weeks with a lower number of births, more newborns are being visually assessed and opposite in weeks with a higher number of births (Figure 3).

## Discussion

Video recordings were feasible and a useful objective tool to evaluate clinical ENC practices in Pemba, Tanzania. The data showed that none of the newborns received the recommended ENC interventions adequately [5]. The study identified omission of key ENC practices including proper thermal care, skin to skin contact and establishment of breastfeeding within the first hour of life and vitamin K administration as well as application of unnecessary practices such as excessive suctioning of breathing newborns.

The findings of the current study are in line with other cross-sectional studies on ENC practices in Ghana [23], Somalia [12], Ethiopia, Kenya, Madagascar, Mozambique, Rwanda and Tanzania [11]. These studies collected data using clinical observations [11,12], vignettes and surveys [23]. Other studies on ENC practices used data obtained from the medical records [13–15] of newborns and their mothers, as well as postpartum interviews with mothers [24]. A large observational study that evaluated six sub-Saharan countries reported that only 45% of newborns were placed skin to skin with their mothers immediately after birth, and only 43% of the mothers were assisted with the initiation of breastfeeding within an hour of their newborn being born. It was demonstrated that, similar to many other sub-Saharan countries, Tanzania faced barriers to the implementation of correct ENC practices [11]. Generally, across all data-collecting methods, a lack of proper thermal care, including inadequate early skin-to-skin contact and the initiation of breastfeeding within one hour of birth did not meet the recommendations [5,11–13,23,24]. However, none of these studies reported exclusively on ENC practices for healthy newborns, dissimilar to the present study.

The use of video recordings in the current study was a major strength. It allowed for a less intrusive way of observation, the generation of less biased data, and it enabled the establishment of a timeline for each newborn for more accurate description of ENC practices. Specifically, indicators like thermal care, routine suctioning, time separated from the mother and observation of the newborns were optimal for video observations, since these were captured within the frame, were subjected to very few or no assumptions, and could not have been performed out of view. Direct observations of delivery care are considered to be the gold standard in low-income settings and to be more reliable than medical record reviews, health staff interviews and patient interviews [11]. The Hawthorne effect is an example of documented bias associated with observational studies with direct observations of clinical care. Healthcare staff may change their behaviour because they are aware that they are being observed, thus resulting in bias [25]. To minimise this effect in the current study, data were collected using anonymous video observations [25]. Previous data have suggested that sustained contact with participants over time improves the quality of data collection and that the Hawthorne effect is mostly observed in the first weeks of observation [26]. When analysing our data in a weekly manner, the only distinct trend, where care practice changes after the initial first weeks is the compliance with breastfeeding possible <1 hour from birth (Figure 3). This indicates that the Hawthorne effect can still be a factor, but since it is only distinct in one care practice the effect might be limited. Another strong argument for using video recordings is the ethical paradox raised by direct (in-person) observation in a delivery room. However, the study had several limitations. Potentially challenging video reviews are based on the judgements of observers, with the associated risk of interobserver variability. However, to overcome potential review bias, the video recordings were analysed using the same review chart; in addition, the video reviews were conducted by the same two researchers, using randomised cross-matching to secure consistency. Secondly, the use of motion triggered cameras and not continuous recordings complicates the exact determination of timing between events, as it skips ahead when no motion is registered. This technical challenge could be overcome with continuous video recording 15 minutes after each motion registered. Another technical challenge was the unstable power supply that required the cameras to run on power banks. The biggest limitation when using video observations is that the observations are limited to what is captured within the frame. The video analyses did therefore not include all the WHO recommended essential newborn care practices. Stimulation must be considered the most imprecise and most biased indicator. To diminish this bias and limitation the study was compared with data from the postnatal questionnaires. Likewise, are the proxy results partially based on assumptions. These were defined based on observations of clinical practice by the research team. One way to overcome this challenge is direct filming of the delivery. This would enable the establishment of an exact timeline of events after birth and eliminate uncertainties about what happens out of view of the camera. For obvious reasons this generates many ethical and controversial dilemmas, especially related to privacy and feasibility.

We had several ethical considerations in using video recordings in a delivery room and the consent process thereof. It could be argued that obtaining consent from a woman while she is in labour (the second stage especially) involves ethical considerations as she may not be in the right state of mind to take in information and provide valid consent. The video inclusion rate (51%) could be affected by the timing of consent, since the consent was obtained during a vulnerable state and that women with late presentation or in an obstetric emergency was not approached. The inclusion rate for the postnatal questionnaire was 81%. The gap between the inclusion rates could be due to the timing of consent since consent for the postnatal questionnaire could be obtained post-delivery. Furthermore, newborns who might have had video consent had no video recording because they were not placed on the resuscitation table after delivery (some were observed to be placed on the scale) or were placed on the resuscitation table together with a newborn without consent and consequently covering of the camera. To improve inclusion rates in future research different placements/tables for newborns with and without consent is recommended.

The most recent (2014–15) service provision assessment report in Tanzania reported better compliance with ENC practices compared to the findings of the current study. Routine care practices, such as early skin-to-skin contact, early initiation of breastfeeding and wrapping newborns, were in place in 90% of the evaluated health facilities [27]. However, the data collected for this report were not based on clinical observations but on interviews with the healthcare staff. The service provision assessment report stated that none of the health facilities in Pemba provided vitamin K or applied antibiotic eye ointment as a routine component of newborn care, which is in line with our results. The administration of both is part of Tanzanian newborn care guidelines [28]

It has been estimated that compliance with newborn care practices immediately after birth prevents 10% of all neonatal mortality [3]. A large 2018 study reported a neonatal mortality rate of 16 per 1,000 live births in Pemba. In Zanzibar, a stillbirth rate of 59 per 1,000 was reported; hence perinatal mortality significantly exceeded the SDG goal [29], which emphasizes the need to improve ENC practices and implement measures to mitigate co-morbidities and mortality in newborns in Pemba. Hypothermia during the newborn period is a widely recognised risk factor for neonatal mortality and morbidity [7,9], also in tropical climates, and in healthy newborns [7]. Similarly, a lack of early breastfeeding has been identified as a risk factor for neonatal mortality [8,21]. The current study found that, on average, newborns spent 25 minutes and 15 seconds separated from their mother, and nearly half of them were insufficiently covered during this time. In the current study, a substantial number of newborns were left unattended within the first hour of life. This can significantly increase the risk of poor thermoregulation and nutrition [30], and, most importantly, delay the ability to observe clinical danger signs. The study team found that only 8% of newborns were observed on video to be stimulated correctly. This could be the result of newborns being stimulated while on their mothers’ abdomen as part of the immediate drying of the newborn and therefore not fully captured on video. This is partially supported by postnatal questionnaires with reports of 113 (38%) newborns being stimulated. Practices that might constitute mistreatment, such as holding the newborn upside down or by one leg or slapping him or her [31] were observed in some videos. Some ENC practices such as administration of vitamin K was not observed possible due to scarcity of supplies as stated in the national service provision assessment report [27]. This and the observed change in a care practice based on the volume of births (Table 3) emphasizes the importance of contextual factors in provision of quality of care.

The recommendation of early initiation of breastfeeding is supported by the findings of several studies [8–10]. Globally, it has been estimated that 43% of all newborns breastfeed within an hour of birth [32]. The current study found that 10% of the newborns spent more than one hour separated from their mother; thus, it was not possible to achieve this care practice goal. The remaining 90% of newborns could have potentially started breastfeeding within an hour of birth, which would have placed Pemba ahead of global estimates.

Our findings identify educational and contextual factors contributing to challenges in provision of quality of ENC. There is consensus that ENC training for healthcare staff improves practices and compliance [14,15,33]. A study from Tanzania reported an increase (i.e. from 39% to 73%) in the overall quality of newborn care post the intervention [30]. Improvements in ENC practices were shown to significantly reduce the rate of newborn intensive care unit admission, with a reduction of both hypothermia and sepsis cases in a large study from Vietnam [14]. A cluster randomised trial conducted in the Democratic Republic of Congo concluded that training birth attendants in ENC skills helped to reduce perinatal mortality [34]. Research into the advantages of ENC practices and healthcare staff training verified that improving ENC skills assisted with a reduction in neonatal mortality. Gaps in ENC practices and the documented effects of healthcare staff training means that continued investment in building the capacity of health staff remains a priority.

The current study demonstrated that ENC compliance was inadequate in this setting at a district hospital in Pemba. An implication for future research is the need to investigate barriers in provision of essential low-cost practices in low-income settings and to consider the impact of possible contextual factors in this regard, such as a scarcity of supplies, a lack of human resources, local cultural practices, and religion. In addition, determining health staff knowledge of the importance of adequate ENC practices should be undertaken.

To the best of our knowledge, this study is the first to have used video recordings to assess the compliance and quality of ENC practices for healthy newborns. Previous studies have used video recordings to analyse newborn resuscitation scenarios. Given the obvious disparity more studies should be conducted using video recordings to evaluate ENC practices to validate methodologies. Video recordings may be feasible and acceptable in LMIC, however further research is needed of using video recording for quality improvement.

## Conclusion

The current study demonstrated using video observations of ENC practices that none of the included newborns received all the recommended ENC interventions. Further research is needed in barriers to provision of essential newborn care in a low resource setting.

## Paper context

Previous clinical observational studies have identified gaps in the quality of ENC practices in low resource settings. This study showed that video observations were a source of valuable objective information and minimize observational bias. The results show that there is substantial room for improvement in the implementation of ENC practices and further research is needed of using video recordings for quality improvements in low resource settings.

## Appendix 1 – Checklist for video analysis

The video analyzing method is created based upon WHO recommendations of essential newborn care (quality indicators), with a matching film indicator and code. While analyzing the data, we will look for film indicators and record the appropriate code with timestamp.

**Table:**

Quality indicators	Film indicators	Film indicators subdivided	Code
Lie the newborn down on a flat and firm surface, wrap it in a dry towel and cover the head to keep it warm.	Newborn wrapped in kanga and head covered	Not covered start time	1AA
		Not covered end time	1AB
		Not covered total time	1AC
		Fully covered	1B
		Uncovered while on table start time	1CA
		Uncovered while on table end time	1CB
		Uncovered total time	1CC
Stimulate the newborn thoroughly by rubbing its back.	Newborn stimulated		2
Avoid separating mother from newborn whenever possible	Separation time = total time on resuscitation table	Separation time	3
Keep warm by skin to skin contact with the mother	Separation time = total time on resuscitation table	Separation time	3
Check breathing and color every 15 minutes	Newborn assessed	Visually assessed start time	4AA
		Visually assessed end time	4AB
		Visually assessed total time	4AC
		Clinical examination start time	4 BA
		Clinical examination end time	4BB
		Clinical examination total time	4BC
		Assessed with stethoscope start time	4CA
		Assessed with stethoscope end time	4CB
		Assessed with stethoscope total time	4CC
Check the cord for bleeding every 15 min, keep it clean and dry.	Cord checked		5
If bleeding, retie it.	Retied if bleeding		6
If the baby is born with meconium or if secretions are blocking the mouth and nose, and you need to resuscitate first clear the airway;	Suction of newborns	Oral suction	7A
		Nasal suction	7B
	Suction of newborns	Suction time start	8AA
		Suction time end	8AB
		Suction time total	8AC
It is important that you SUCTION meconium OUT of the newborn!	Correct suctioning technique	Yes	9A
		No	9B
		Uncertain	9C
Give 1 mg vitamin K (IM)	Intramuscular administration of vitamin K		10
Start breastfeeding within the first hour after birth and let the newborn feed on demand.	Separation time from mother>1 hour		11
Apply antibiotic eye ointment	Application of antibiotic ointment		12
